# Bioconversion of vitamin D_3_ to bioactive calcifediol and calcitriol as high-value compounds

**DOI:** 10.1186/s13068-022-02209-8

**Published:** 2022-10-13

**Authors:** Zheyi Wang, Yan Zeng, Hongmin Jia, Niping Yang, Mengshuang Liu, Mingyue Jiang, Yanning Zheng

**Affiliations:** 1 State Key Laboratory of Microbial Resources, Institute of Microbiology, Chinese Academy of Sciences, No.1 Beichen West Road, Chaoyang District, Beijing, 100101 China; 2 University of Chinese Academy of Sciences, No.19A Yuquan Road, Beijing, 100049 China; 3 China Animal Husbandry Industry Co. Ltd, Beijing, 100095 China; 4 School of Life Sciences, Hebei University, No. 180 Wusi Dong Road, Baoding, 071002 China

**Keywords:** Vitamin D_3_, Calcifediol, Calcitriol, Bioconversion, Cytochrome P450s

## Abstract

Biological catalysis is an important approach for the production of high-value-added compounds, especially for products with complex structures. Limited by the complex steps of chemical synthesis and low yields, the bioconversion of vitamin D_3_ (VD_3_) to calcifediol and calcitriol, which are natural steroid products with high added value and significantly higher biological activity compared to VD_3_, is probably the most promising strategy for calcifediol and calcitriol production, and can be used as an alternative method for chemical synthesis. The conversion efficiency of VD_3_ to calcifediol and calcitriol has continued to rise in the past few decades with the help of several different VD_3_ hydroxylases, mostly cytochrome P450s (CYPs), and newly isolated strains. The production of calcifediol and calcitriol can be systematically increased in different ways. Specific CYPs and steroid C25 dehydrogenase (S25DH), as VD_3_ hydroxylases, are capable of converting VD_3_ to calcifediol and calcitriol. Some isolated actinomycetes have also been exploited for fermentative production of calcifediol and calcitriol, although the VD_3_ hydroxylases of these strains have not been elucidated. With the rapid development of synthetic biology and enzyme engineering, quite a lot of advances in bioproduction of calcifediol and calcitriol has been achieved in recent years. Therefore, here we review the successful strategies of promoting VD_3_ hydroxylation and provide some perspective on how to further improve the bioconversion of VD_3_ to calcifediol and calcitriol.

## Background

Vitamin D_3_ (VD_3_) is an essential steroid hormone in the human body and a fat-soluble prohormone. In addition to being obtained from food, VD_3_ can be synthesized in the human body by ultraviolet irradiation using 7-dehydrocholesterol as a precursor. VD_3_ has an important role in regulating calcium and phosphorus metabolism and controlling the growth and development of bone cells [1]. However, the biological activity of VD_3_ is relatively low compared with that of its active forms. In the human body, two-step hydroxylation under the catalysis of cytochrome P450s (CYPs) is required for its full biological activity. VD_3_ is first converted to 25(OH)VD_3_ (calcifediol) in the liver, mainly but not exclusively by CYP2R1, and then further converted to 1α,25(OH)_2_VD_3_ (calcitriol) by CYP27B1 in the kidney to gain its full activity [2]. VD_3_ helps treat osteoporosis, chronic renal dysfunction and other diseases [3, 4]. Supplementation with the hydroxylation products of VD_3_ instead of VD_3_ itself eliminates the need for the hydroxylation process in the human body, making it important for patients with abnormalities in vitamin D metabolism. It has been reported that supplementation with calcitriol is helpful for the treatment of liver [5] and kidney disorders [6], and calcifediol is used for the treatment of calcium disorders, such as rickets [7]. In addition, calcifediol or calcitriol supplementation is able to prevent cartilage diseases, improve egg production and egg quality of laying hen, and enhance reproductive performance of breeding swine in the livestock and poultry production [8–10].

At present, calcifediol and calcitriol are mainly produced by chemical synthesis, which requires complex protection and deprotection steps for the regioselective introduction of hydroxyl groups, especially for hydroxylation on C-1, limiting the industrial production [11, 12]. For example, starting with hydrindenylpropanol, Lythgoe et al. [13] synthesized des-AB-cholestane-8β,25-diol, which could be further used as a substrate for the synthesis of calcifediol. The synthesis process of des-AB-cholestane-8β,25-diol requires multiple steps and further conversion to calcifediol. Calcitriol can be obtained by condensation of 1α-hydroxylated phosphine oxide with 25-hydroxy ketone [14]. However, the substrates also require multiple steps for synthesis. Thus, compared with chemical synthesis, the bioconversion of VD_3_ is a successive process that comprises two (25- and 1α) or one (only 25-) hydroxylation steps. In addition, the yield of chemical synthesis of calcifediol is low (the classical approach by photochemical ring opening of steroidal Δ^5,7^-dienes yields less than 1%) [15]. Therefore, increasing attention is being paid to the bioconversion of VD_3_ to calcifediol and calcitriol. Though hydroxylated sterols could be synthesized by transgenic *Arabidopsis* plants with non-heme monooxygenase [16], VD_3_ has not yet been reported as a substrate. Microorganisms have been a powerful tool for VD_3_ hydroxylation research, and in some work, the VD_3_ conversion rate was remarkable. Therefore, a bioconversion process with VD_3_ as the substrate and microorganisms as the chassis cells is promising for its simplicity and low pollution.

In the past few decades, a large number of strains expressing VD_3_ hydroxylases have been developed for VD_3_ bioconversion. A common strategy is to screen organisms with VD_3_ hydroxylation capabilities and then examine their calcifediol and calcitriol production abilities by fermentation. Furthermore, the characterization of VD_3_ hydroxylases from different sources and their heterologous expression in different host cells are also topics of particular interest in this field. These VD_3_ hydroxylases mainly refer to cytochrome P450s. In addition, steroid C25 dehydrogenase (S25DH) was also reported to have the ability to hydroxylate VD_3_ at the C-25 position. However, S25DH is sensitive to oxygen, causing a high cost for calcifediol production [17]. CYPs are the primary enzymes currently used for bioconversion of VD_3_ to its active forms. A variety of CYPs from mammals and bacteria (mainly from actinomycetes) that are capable of converting VD_3_ to calcifediol and calcitriol have been identified, and highly active mutants have been constructed. Enhanced calcifediol and calcitriol production could further benefit from the rapid development of creating new enzymes and new biochemical reactions via computational redesign of enzymes.

In general, enzymes and whole cells are two categories of biocatalysts commonly used for bioconversion [18, 19]. Here, we review advances in the characterization and engineering of VD_3_ hydroxylase that plays a key role in VD_3_ bioconversion, and progress in whole-cell bioconversion, which can use inexpensive and abundant raw materials and avoiding the addition of expensive cofactors.

## VD_3_ hydroxylases and their protein engineering

**Fig. 1:**
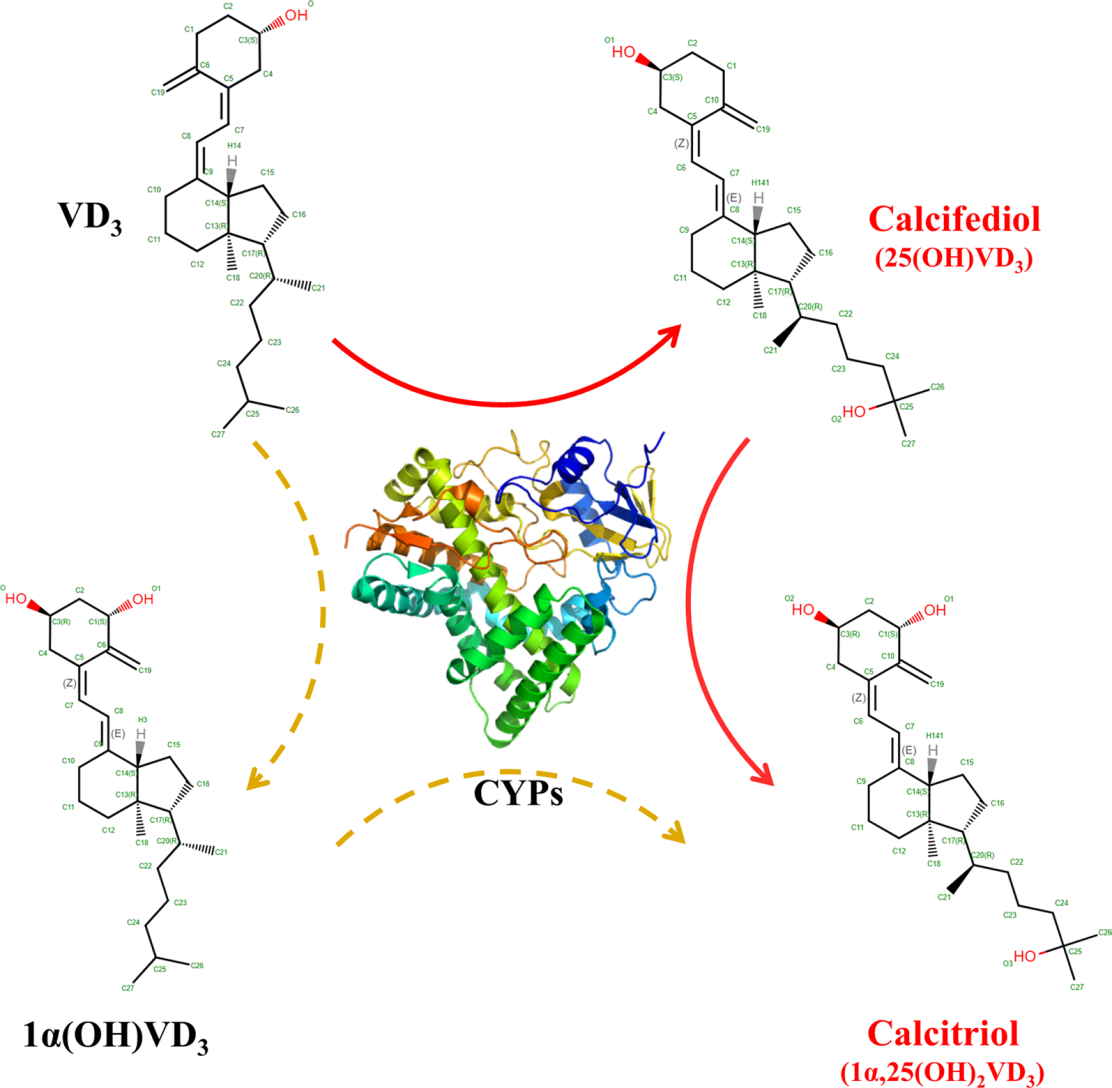
VD_3_ bioconversion process. VD_3_ is sequentially converted to calcifediol and calcitriol by CYPs. Some uncommon CYPs can also catalyze the conversion of VD_3_ to calcitrol with 1α(OH)VD_3_ as the intermediate. Many CYPs, such as CYP27B1, can catalyze hydroxylation at two sites, as they have relatively wide substrate specificity and regioselectivity

For the synthesis of complex compounds, including calcifediol and calcitriol, enzymatic conversion has high regio- and stereoselectivity when compared with chemical synthesis. VD_3_ hydroxylases, most of which are CYPs, play a central role in the bioconversion of VD_3_ into calcifediol and calcitriol. Cytochrome P450s (CYPs) are a class of heme-containing enzymes with similar structures. CYPs catalyze a variety of reactions, mostly monooxygenation reactions, and have a wide range of substrate specificities [20]. The main CYPs related to VD_3_ hydroxylation are shown in Table 1 and Fig. 1.

**Table 1:** CYPs from different organisms and their substrate specificities

CYPs	Species	Substrates	Products	References
Mammalian CYPs				
CYP27A1	*Homo sapiens* *Rattus norvegicus* (rat)	VD_3_ Calcifediol	Calcifediol Calcitriol	[24]
CYP27B1	*Mus musculus* (mouse)	VD_3_, Calcifediol	Calcifediol 1α(OH)VD_3_ Calcitriol	[23]
CYP2D25	*Susscrofa domestica* (pig)	VD_3_ Calcifediol	Calcifediol Calcitriol	[35]
CYP2J2	*Homo sapiens*	VD_3_	Calcifediol	[36]
CYP2J3	*Rattus norvegicus*	VD_3_	Calcifediol	[27]
CYP2C11	*Rattus norvegicus*	VD_3_	Calcifediol	[37]
CYP2R1	*Homo sapiens*	VD_3_	Calcifediol	[28]
Bacterial CYPs				
CYP105A1	*Streptomyces griseolus*	VD_3_ Calcifediol	Calcifediol Calcitriol	[41]
CYP105A2	*Pseudonocardia autotrophica*	VD_3_	Calcifediol	[40]
CYP107CB2	*Bacillus lehensis*	VD_3_ Calcifediol	Calcifediol Calcitriol	[42]
CYP107BR1	*Pseudonocardia autotrophica*	VD_3_ Calcifediol	Calcifediol Calcitriol	[48]

Mammalian and bacterial CYPs have been widely utilized for the bioproduction of calcifediol and calcitriol, the active forms of VD_3_. Mammalian CYPs have attracted great attention as they are associated with VD_3_ metabolism related diseases. Bacterial CYPs are also of great importance because of their enormous functional diversity. A unique property of bacterial CYPs is that carbon monoxide (CO), SKF-525-A and metyrapone inhibit the hydroxylase activities of bacterial CYPs. Therefore, reduced CO difference spectroscopy can be used to identify bacterial CYPs that function as VD_3_ hydroxylases [21, 22]. To simplify the purification of CYPs, especially mammalian CYPs, the heterologous expression of CYPs in model organisms can increase protein yield and reduce the cost [23]. *Escherichia coli* and yeast are ideal host cells for heterologous expression of CYPs, as they grow quickly and genetic tools for their modifications have been well developed. Given that the hydroxylation reaction of CYPs requires redox proteins to deliver electrons, it is of great importance to improve VD_3_ conversion rates using appropriate redox partners and coenzyme regeneration systems. Because of the existence of abundant redox proteins, actinomycetes are also becoming popular for heterologous expression of VD_3_ hydroxylases. With an increased understanding of the relationship between the protein structures of CYPs and their hydroxylation abilities, protein engineering has been widely applied to the evolution of CYPs in the laboratory, which aims to improve the activity of CYPs for enhanced VD_3_ bioconversion.

### CYPs from mammals

Mammalian VD_3_ hydroxylases such as CYP27A1, CYP27B1, CYP2D25, CYP2J3 and CYP2R1 have been found to be able to catalyze C-25 or C-1α hydroxylation [24–28]. The CYP27A1 was the first mammalian CYP to be thoroughly studied. *Saccharomyces cerevisiae* expressing a modified rat CYP27A1 localized in microsomes could convert 1α(OH)VD_3_ to calcitriol when adrenodoxin (ADX) and NADPH-adrenodoxin reductase (ADR) were coexpresseded [24]. The ADRs from yeast and rats were unable to transfer electrons for rat CYP27A1. However, the purified bovine ADR and ADX facilitated the reaction. Therefore, the mitochondria-targeting signals of bovine ADR and ADX were removed to construct an electron transfer chain between the cytoplasm and the modified CYP27A1 that was incorporated into microsomes. The modified rat CYP27A1 could convert 1α(OH)VD_3_ to calcitriol with the help of the newly constructed electron transfer chain. In one study, human CYP27A1, which also belongs to the CYP27 family was expressed in *E. coli* to examine its enzyme activity with VD_3_, and only a low activity of human CYP27A1 toward VD_3_ (with *V*_max_ of 0.27 mol/min/mol P450) was observed in this reaction system [29]. Although the 25-hydroxylation activity of human CYP27A1 was notably lower than that of several other mammalian CYPs such as human CYP2R1 (*k*_cat_ = 0.61 min^−1^ for 25-hydroxylation activity toward vitamin D_3_ with CYP27A1 compared to *k*_cat_ = 0.27 min^−1^ with CYP2R1), human CYP27A1 could perform 1α-hydroxylation toward calcifediol. Besides calcifediol and calcitriol, other products of human CYP27A1 hydroxylation include 26(OH)VD_3_, 24R,25(OH)_2_VD_3_, 27(OH)VD_3_, 25,26(OH)_2_VD_3_, 25,27(OH)_2_VD_3_, 27-oxo-VD_3_ and a dehydrogenated form of VD_3_, making the recovery of calcifediol or calcitriol much more difficult. In addition, ergosterol in the yeast membrane might inhibit the VD_3_ bioconversion as a potential substrate for CYP27A1. Therefore, compared with yeast, *E. coli* may be a better host for heterologous expression of mammalian CYPs due to its lack of the P450 genes and steroids [30].

Mitochondrial CYP27A1 in human liver is considered to be one of the CYPs that performs C-25 hydroxylation, while CYP27B1, another member of the CYP27 family, is responsible for hydroxylation at the C-1α position. However, Uchida et al. found that, by adding purified ADR and ADX to the reaction system, mouse CYP27B1 could perform both 25-hydroxylation and 1α-hydroxylation, similar to CYP27A1, suggesting that 1α-hydroxylation and 25-hydroxylation of vitamin D_3_ are closely related [23]. This observation led to the hypothesis that vitamin D_3_ can be arranged in the opposite direction of the substrate-binding pocket of CYP27B1. In addition, molecular chaperone GroEL/ES greatly enhanced the expression of mouse CYP27B1 in *E. coli*, making great progress for heterologous expression of CYPs in the future. In addition to mouse CYP27B1, human CYP27B1 also has 1α-hydroxylation activity [25]. However, the variant human CYP27B1 with CYP27B1^T409I^ or CYP27B1^Q65H^ amino acid substitution caused vitamin D 1α-hydroxylase deficiency (Vitamin D-dependent Rickets Type 1). The identification of amino acids that interact with the substrate could provide structural clues for the protein engineering of CYPs. The Ser^408^ residue in mouse CYP27B1 and the Thr^409^ residue in human CYP27B1 were found to be responsible for the C-1α hydroxylation by forming a hydrogen bond with the 25-hydroxyl group of calcifediol [31]. However, the problem with the low expression of human CYP27B1 still exists. Different strategies were thus employed to optimize the expression of human CYP27B1. High expression of human CYP27B1, shown by conversion to calcitriol with a yield of above 6000 pmol/mg protein in 30 min, was finally achieved by coexpressing GroEL/ES and human ADX/ADR rather than bovine ADX/ADR [32]. The addition of cardiolipin to the reconstituted system markedly lowered (threefold) the *K*_m_ of human CYP27B1 for calcifediol, suggesting that the activity of CYPs could also be influenced by phospholipid composition.

Several other mammalian CYPs were also found to be relevant to VD_3_ hydroxylation. Both human CYP2R1 and human CYP27A1 can catalyze the 25-hydroxylation of VD_3_. However, the 25-hydroxylation activity of CYP2R1 on VD_3_ is five times higher than that of CYP27A1. In addition, a mutation in the human *CYP2R1* gene results in vitamin D-dependent rickets [33]. These data indicate that CYP2R1 also plays an important role in calcifediol biosynthesis in human body [28]. The GroEL/ES chaperonin can also be used to increase the expression level of human CYP2R1 in *E. coli* [34]. To understand which amino acid residues are responsible for the substrate specificity of CYP2R1, the crystal structure of CYP2R1 in complex with VD_3_ was solved. VD_3_ binds in an elongated conformation with the aliphatic side-chain pointing to the heme. The substrate access channel is covered by the ordered B′-helix. The conserved active site in a closed conformation of CYP2R1 is mainly composed of hydrophobic amino acid residues. Porcine CYP2D25 was expressed in yeast by Hosseinpour [26] and Araya [35], and the substitution of five residues in substrate binding site 3 (SRS-3) inhibited its 25-hydroxylase activity. Besides 25-hydroxylase towards VD_3_, CYP2D25 is also able to convert calcifediol into calcitriol, 25,26(OH)_2_VD_3_, and 25,27(OH)_2_VD_3_.

Just like CYP2R1 as VD_3_ 25-hydroxylase, the recombinant rat CYP2J3 shows a 25-hydroxylation activity when it was coexpressed with GroEL/ES in *E. coli* [27]. The turnover numbers were 3.3 and 22 toward VD_3_ and 1α(OH)VD_3_, respectively. Human CYP2J2 shows 73% amino acid homology with CYP2J3. After the deletion of a putative membrane anchor region in the N-terminus and the coexpression of GroEL/ES, efficient expression of CYP2J2 was achieved. However, human CYP2J2 does not seem to be a principal VD_3_ 25-hydroxylase [36]. The CYP2C11, a male-specific hepatic recombinant microsomal vitamin D 25-hydroxylase, has a substrate preference toward VD_3_ over 1α(OH)VD_3_ [37]. The 25-hydroxylation activity of the CYP2C11 towards VD_2_ was higher than that towards VD_3_ (25-hydroxylation rate of 0.49 ± 0.03 nmol·nmol^−1^·1.5 h^−1^ for VD_2_ compared with 0.07 ± 0.02 nmol·nmol^−1^·1.5 h^−1^ for VD_3_). The examples of CYP2J2 and CYP2C11 imply that some CYPs from mammals take VD_2_ as a prioritized substrate and thus might not be the best choice for the hydroxylation of VD_3_.

### CYPs from microorganisms

CYPs existing in a variety of organisms are involved in many biosynthetic pathways [20, 25]. Since the initial discovery of the VD_3_ hydroxylation in *Amycolata sp.* and *Streptomyces sp.* strains, CYPs from actinomycetes have been widely used for the VD_3_ bioconversion process [21, 22, 38, 39]. The 25-hydroxylation activity of VD_3_ by CYP105A2 (with the highest yield of 10%) was observed by heterologous expression of CYP105A2 from *Amycolata autotrophica* (*Pseudonocardia autotrophica*) in *Streptomyces lividans* [40]. Cytochrome P450SU-1 (CYP105A1) from *Streptomyces griseolus* was found to convert VD_3_ to 25(OH)VD_3_ when expressed in *E. coli* [41]. Although the amino acid sequences of CYP105A1 and CYP105A2 share 55% identity, there is a clear difference in catalytic activity between CYP105A1 and CYP105A2. Besides the 25-hydroxylation of VD_3_, CYP105A1 also shows 1α-hydroxylation of calcifediol. In addition, CYP105A1 can also catalyze the hydroxylation of VD_2_ with a relatively high *V*_max_/*K*_m_ value (0.142 L^−1^·min^−1^/mol P450, compared to 30 L^−1^·min^−1^/mol P450 for VD_3_). The CYP107CB2 from *Bacillus lehensis* G1 was classified into the CYP107 subfamily and showed 25-hydroxylation activity of both VD_3_ and 1α(OH)VD_3_ [42].

**Fig. 2:**
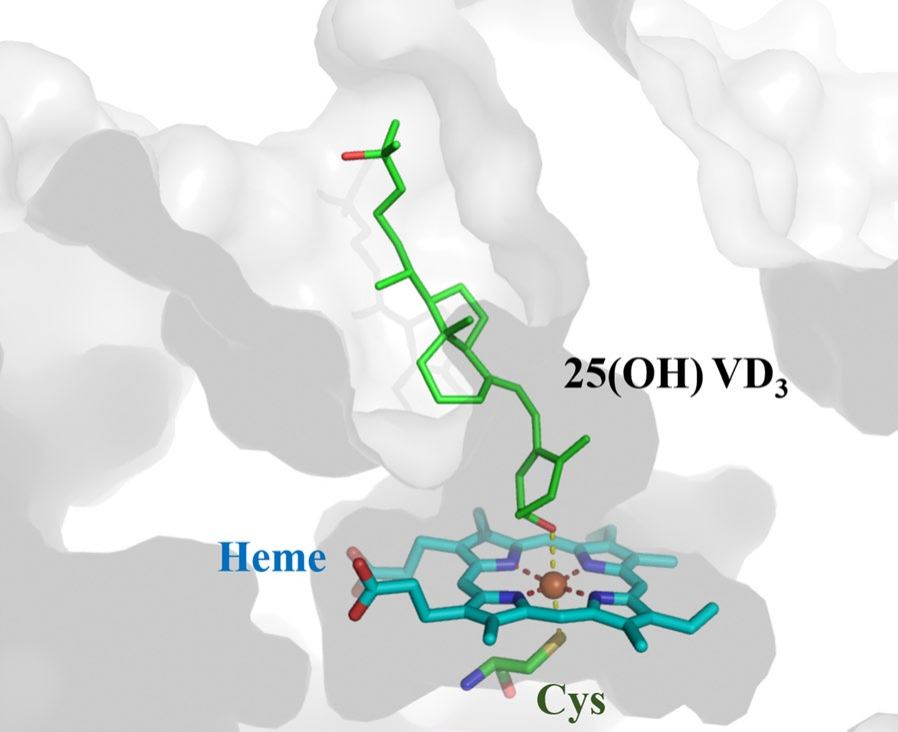
Schematic representation of the substrate binding pocket. The substrate calcifediol is located in the substrate binding pocket in a suitable position, and will be further catalyzed by the Cys-linked heme

CYPs that catalyze the hydroxylation of VD_3_ share a common catalytic mechanism with most CYPs. The catalytic cycle occurs on the heme prosthetic group which is linked by a conserved cysteine [43], but the catalytic specificity of CYPs mainly depends on their substrate binding pockets [44] (Fig. 2). Based on the catalytic mechanism, the catalytic specificity of CYP for VD_3_ can be improved by directed evolution of CYPs.

Structural analysis of CYP105A1 in complex with calcitriol found that Arg^73^ and Arg^84^ along with Arg^193^ were located in the distal pocket of CYP105A1. The Arg^73^ and Arg^84^ may have an inhibitory effect on activity, while Arg^193^ is required for the activity [39, 45, 46]. Therefore, a series of CYP105A1 variants focusing on residues Arg^73^ and Arg^84^ were made to achieve a higher activity (with relative *k*cat/*K*_m_ compared with wild type ranging from 1 min^−1^·μM^−1^ to 43 min^−1^·μM^−1^ with 1α(OH)VD_3_ and calcifediol as substrate). Compared with the wild-type CYP105A1, the activity of CYP105A1^R84A^ variant greatly increased, with activity of 25-hydroxylation toward 1α(OH)VD_3_ and 25-hydroxylation toward calcifediol are approximate 27-fold and 16-fold than CYP105A1^WT^, respectively. The improved activity can be attributed to the loss of two hydrogen bonds, which resulted in a new transient binding site for both the substrate and product [45]. The variant of CYP105A1 containing amino acid substitutions R73V and R84A exhibited much higher hydroxylation activities at positions C-1α and C-25 compared to the wild type; the CYP105A1^R84A^ variant also shows a *k*cat/*K*_m_ value for 25-hydroxylation that is 435-fold higher than that of the wild type; the CYP105A1^R84A^ mutant also shows a higher 1α-hydroxylation capability than any single mutant. The amino acid residue at position 73 affects the location and conformation of the substrate in the reaction center, while the amino acid residue at position 84 affects the location and conformation of the transient binding site. In addition, the CYP105A1^R73VR84A^ [39] also catalyzes the hydroxylation at position C-26, with 1α,25(R),26(OH)_3_VD_3_ and 1α,25(S),26(OH)_3_VD_3_ as products. The presence of C-26 hydroxylation activity could be caused by the rotation of a secosteroid skeleton and an evident conformational change of the side chain in the heme pocket.

Inspired by the engineering of CYP105A1^R73VR84A^, arginine residues around the substrate entrance and active site of CYP105D7 were mutated to generate a double mutant CYP105D7^R70AR190A^ [47]. The CYP105D7^R70AR190A^ achieved an almost ninefold increase in the conversion rate of testosterone, suggesting that the arginine residues around the substrate entrance and active site play important roles in determining the functions of the CYPs. Interestingly, the CYP105A1 is not the only VD_3_ hydroxylase found in *P. autotrophica*. Vdh (CYP107BR1) from *P. autotrophica* NBRC 12743 also has the capability of converting VD_3_ into calcifediol and calcitriol [48]. Different from other VD_3_ hydroxylases, the Vdh has no preferred regio-specific hydroxylation of VD_3_.

A Vdh variant designated as Vdh-K1 with four amino acid substitutions was obtained by means of random mutagenesis, with its activity an order of magnitude higher than that of wild-type Vdh. Yasutake et al. then analyzed the structures of Vdh and Vdh-K1 [49]. The wild-type Vdh showed an open conformation, and the distal heme pocket was exposed to solvent with or without substrate. In contrast, Vdh-K1 shows a closed conformation, increasing the substrate binding affinity and catalytic activity. In addition, the combination of VD_3_ and calcifediol with Vdh-K1 is in an antiparallel orientation. It is worth noting that the four substituted residues of Vdh-K1 are dispersed throughout the protein but result in a closed conformation of the heme pocket. The Vdh^T107A^ variant, which was made by modifying the putative ferredoxin-binding site of Vdh, also exhibited a closed conformation similar to Vdh-K1 bound to VD_3_ [50]. The ferredoxin-binding surfaces of Vdh^T107A^ seem to show a stronger positive potential that is conducive to the binding of ferredoxin when compared with the structures of Vdh-K1. Although the enzyme activity of Vdh-K1 was much higher than that of wild-type Vdh, its expression level in *Rhodococcus erythropolis* cells was low, in contrast with that of Vdh^T107A^, which was approximately 70% of Vdh^WT^. In addition, Vdh^T107A^ has an improved activity that is comparable to that of Vdh-K1, and achieves a similar expression that is comparable to Vdh simultaneously.

The above examples are the modification of the substrate binding pocket. Nevertheless, the engineering technology of CYPs has been greatly developed. Drawing on these technologies, it may be possible to use allosteric regulation, decoy-based small molecules etc., for reference in the hydroxylation of VD_3_ in the future. For example, the conformational transition of CYP3A4 is induced by the allosteric ligand progesterone (PGS), and CYP3A4-PGS conjugates thus acquire a twofold higher capacity to oxidize testosterone than CYP3A4 [51]. Inert decoy molecules that have a native substrate-like structure, can constrain substrates, such as benzene and benzene derivatives, into the suitable positions to facilitate the catalysis by targeting the enzyme substrate pocket to induce an active enzyme intermediate [52, 53]. If the above technologies are combined into the catalytic reaction of VD_3_, it may be possible to expand the types of CYPs that can be used to catalyze VD_3_, inspiring future research that may have unforeseen benefits.

### Conversion of VD_3_ by steroid C25 dehydrogenase

Rugor et al. reported that steroid C25 dehydrogenase (S25DH) from the β-proteobacteria *Sterolibacterium denitrificans* was able to catalyze the regioselective hydroxylation of sterols and their derivatives [17]. The reaction was carried out in a batch reactor supplemented with purified S25DH (as the enzyme), VD_3_ (substrate), hydroxypropyl-β-cyclodextrin (solubilizer), 2-methoxyethanol (organic cosolvent) and K_3_[Fe(CN)_6_] (electron acceptor). A calcifediol titer of 1.4 g/L was achieved after a 162-h incubation, with a yield as high as 99%.

Compared with CYPs, S25DH has advantages as well as disadvantages. The catalytic activity of S25DH is higher than that of the CYPs reported thus far. In addition, the regioselectivity toward the C-25 position of VD_3_ is higher in S25DH than that in CYPs, making the recovery of calcifediol much easier. However, S25DH is sensitive to oxygen, so the bioconversion process must be carried out under anaerobic conditions [54]. Jacoby et al. developed a platform for the overproduction of four steroid C-25 hydroxylases (S25DH_1_ and three isoenzymes S25DH_2_, S25DH_3_, and S25DH_4_) by coexpressing an essential chaperone in the betaproteobacterium *Thauera aromatica* K172 [55]. The crude extract from *T. aromatica* that overexpresseed S25DH_1_ achieved a reaction rate that was 6.5-fold higher than that of the wild-type bacterial extract. However, the oxygen intolerance and high cost prevent S25DH from being applied for the industrial production of calcifediol.

## Whole cell conversion of VD_3_ into calcifediol and calcitriol

A whole-cell conversion system expressing the CYPs of interest has advantages over enzymatic conversion. It avoids adding the expensive cofactor NAD(P)H, and the hydrogen dioxide produced by the uncoupling reactions does not accumulat because of the presence of catalase in the cell. Therefore, increasing attention has been given to the whole cell conversion of VD_3_ into calcifediol and calcitriol. initially, attempts were made to screen natural microbial strains that are able to convert VD_3_ to calcifediol and calcitriol in nature. With the development of genome editing and synthetic biology, the production of calcifediol and calcitriol has been further improved by either overexpressing heterogenous VD_3_ hydroxylase genes or altering the electron transfer chains.

### Conversion of VD_3_ to calcifediol and calcitriol by microbes isolated in nature

Sasaki et al. reported the bioconversion of VD_3_ derivatives for the first time [21]. After screening approximately 300 *Streptomyces* strains, *S. sclerotialus* FERM BP-1370 and *S. roseosporus* FERM BP-1574 were found to be able to introduce hydroxy group at C-1α position of calcifediol and C-25 position of 1α(OH)VD_3_, respectively. Although VD_3_ derivatives were used as substrates rather than VD_3_ itself, this work demonstrated that microorganisms could be used for the production of hydroxylated derivatives of VD_3_. By further screening within bacterial and fungal strains, *P. autotrophica* FERM BP-1573 was chosen from twelve VD_3_-hydroxylating strains and achieved a calcifediol titer of 8.3 mg/L and a calcitriol titer of 0.17 mg/L in a 200 L fermenter [22].

With the ever-increasing number of newly discovered VD_3_-hydroxylating strains, the production of calcifediol and calcitriol has been further improved. The actinomycete *Kutzneria albida* produced 70.4 mg/L calcifediol and 2.0 mg/L calcitriol in an optimized conversion process [56]. When using *Pseudonocardia sp*. KCTC 1029BP as a whole-cell catalyst, calcifediol production further rose to 356 mg/L, and calcitriol production rose to 61.87 mg/L, with bioconversion yields of 59.4% and 30.94%, respectively [57, 58]. More recently, Tang et al. obtained up to 830 mg/L calcifediol using *Bacillus cereus* zju 4-2, representing a significant increase in calcifediol production [59] (Table 2). In addition to calcifediol and calcitriol, some side products of VD_3_ hydroxylation such as 2α,25(OH)_2_VD_3_ could also be synthesized, increasing the difficulty of product recovery [60]. It is of great significance to reduce the formation of side products by isolating a microbial strain with high regioselectivity.

**Table 2:** Whole-cell bioconversion of VD_3_ to calcifediol and calcitriol

Strains	Titer (mg/L)	Productivity (mg/L/d)	Yield (g/g)	References
Calcifediol				
* Pseudonocardia autotrophica* FERM BP-1573	8.3	2.6	NA	[21, 22]
* Pseudonocardia sp.* KCTC 1029BP	356	71.2	0.59	[58]
* Pseudonocardia autotrophica* CGMCC5098	639	127.8	0.61	[75]
* Bacillus cereus* zju 4–2	830	415.0	0.42	[59]
* Kutzneria albida*	70	35.2	0.14	[56]
Calcitriol				
* Pseudonocardia autotrophica* FERM BP-1573	0.17	/	NA	[22]
* Pseudonocardia sp.* KCTC 1029BP	62	6.9	0.31	[58]
* Pseudonocardia autotrophica* 100U-19	32	10.7	NA	[60]
* Kutzneria albida*	2	1.0	NA	[56]

Though many attempts have been made to enhance the calcifediol and calcitriol production, the acquirable concentrations of calcifediol and calcitriol in the fermentation broth are not yet satisfactory. Therefore, a series of fermentation parameters have been optimized to further improve the production of calcifediol and calcitriol. The influence of bioconversion buffers, solubilizers, and metal salts on calcitriol production has also been examined, and optimized bioconversion medium was finally been obtained. The optimization of three key variables (aeration rate, resting cell concentration and temperature) in *Pseudonocardia* fermentation improves the VD_3_ bioconversion [57, 58].

### Conversion of VD_3_ to calcifediol and calcitriol by engineered strains

**Fig. 3:**
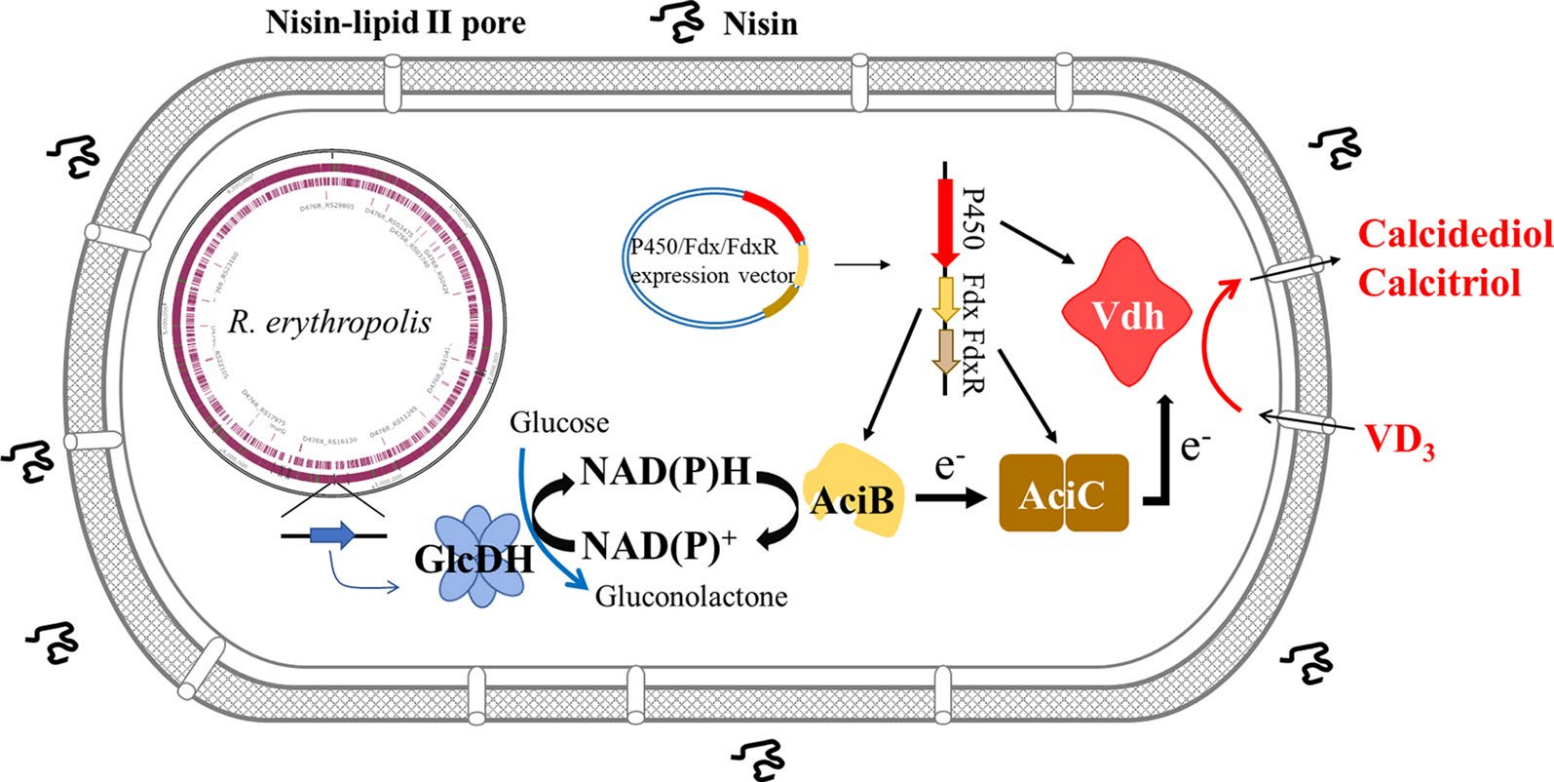
NAD(P)H-regeneration system enhances the bioconversion of VD_3_ to calcifediol and calcitriol in engineered *R. erythropolis*. Sufficient reducing equivalents (NADH/NADPH) obtained by the NAD(P)H-regeneration system (GlcDH from *B. megaterium*) provide more electrons required for the catalysis of the hydroxylation reaction. Moreover, nisin treatment contributes to increased permeability of the cell membrane

**Fig. 4:**
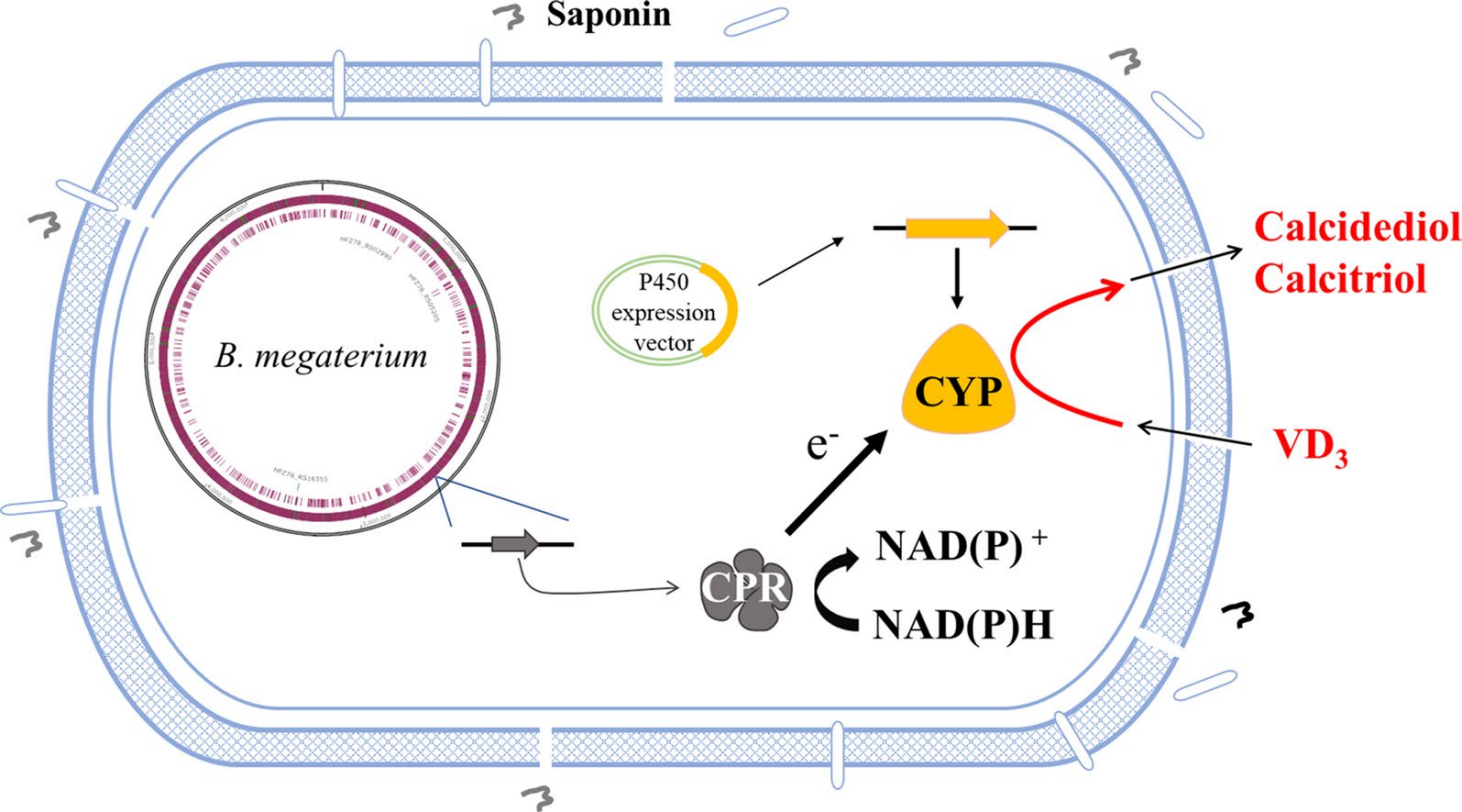
*B. megaterium *is used as a host cell for VD_3_ bioconversion. The electrons required for CYP activity are supplied by the endogenous cytochrome P450 reductase (CPR): NADPH-dependent diflavin reductase [76]. *Quillaja *saponin is used as a membrane-solubilizing agent

With the development of genomics and synthetic biology, engineering of microbial cells provides a new strategy to improve the production of calcifediol and calcitriol. Besides previously mentioned heterologous expression of CYPs for purpose of enzymatic activity characterization and structural analysis, design of engineered strains for whole-cell bioconversion could be applied for industrial-scale production of calcifediol and calcitriol. Actinomycete chassis cells such as *S. lividans* and *R. erythropolis* have stable redox environments, which may explain why most bacterial VD_3_-hydroxylating CYPs have been discovered in actinomycetes; thus, these cells could be promising chassis cells for calcifediol and calcitriol production. When expressing CYP105A1^R73VR84A^ in *S. lividans*, approximately 7.7 mg/L calcifediol was observed in the culture [39]. *R. erythropolis* is another industrially important actinomycete that exhibits resistance to organic solvents, so it was used as a host cell to convert VD_3_ to calcifediol and calcitriol [61] (Fig. 3). The permeability of VD_3_ into the cytoplasm is a rate-limiting step for VD_3_ bioconversion [62]. In addition, the electron carrier ferredoxin and the NADH-regeneration system also play essential roles in the conversion of VD_3_ to its bioactive form. When Vdh was coexpressed with the redox partners AciBC and glucose dehydrogenase GlcDH-IV in nisin-treated *R. erythropolis* cells, 1176.5 μg calcifediol was obtained, representing an improved calcifediol production when compared with the wild-type strain [61]. Deletion of *CYP-sb3* gene dramatically impaired the VD_3_ bioconversion of by *Sebekia benihana*, suggesting that CYP-sb3a functions as a VD_3_ hydroxylase. When CYP-sb3a was expressed in *Streptomyces coelicolor* that originally had no VD_3_ hydroxylation ability, calcifediol and calcitriol were detected in the recombinant *S. coelicolor* cells [63]. *B. megaterium* is another popular host that has been used for VD_3_ bioconversion (Fig. 4). When *B. megaterium* expressing human CYP27A1 was used as whole-cell catalyst, 80.81 mg/L VD_3_ was converted to its bioactive form. In addition, *B. megaterium* overexpressing CYP109A2^T103A^ produced 282.7 mg/L calcifediol in 48 h under optimized conditions. Though there are still technical challenges for calcifediol and calcitriol production by engineered strain, designing better artificial strains is a promising strategy to further enhance the calcifediol and calcitriol production by designing better artificial strains.

With the help of the NADPH-regenerating system, approximately 40% VD_3_ was converted to calcifediol in 3 hours. The CYP109A2 [64] and CYP109E1 [65] from *Bacillus megaterium* also exhibited high VD_3_ bioconversion abilities. After 24 h of whole-cell catalysis, the conversion rates of CYP109A2 and CYP109E1 reached 76% and 95%, respectively. However, NMR analysis showed that CYP109E1 catalyzed the hydroxylation of VD_3_ at both C-24 and C-25. Approximately twofold more calcifediol was produced in the CYP109E1^I85A^ variant obtained by site-directed mutation than in the wild-type CYP109E1 through whole-cell bioconversion, suggesting that the CYP109E1^I85A^ variant achieves improved selectivity for 25-hydroxylation of VD_3_. In addition, after a 48-h incubation under optimized conditions, 282.7 mg/L calcifediol was produced by CYP109A2^T103A^, a more efficient variant obtained by protein engineering of CYP109A2 [66].

The highest yield by engineered strains was achieved by the protein engineering of Vdh (CYP107BR1). For the Vdh and its variants, when *R. erythropolis* cells expressing Vdh^T107A^ were treated with nisin for VD_3_ conversion, 573 mg/L calcifediol was produced in 2 hours, representing a significantly improved bioconversion rate. Compared with wild-type Vdh, a 1.7-fold of calcifediol production was observed in shorter time (2 h compared with 16 h).

Whether by isolated strains or engineered strains, the efficiency of the VD_3_ bioconversion process is limited in different levels, mainly genes expression, enzyme activity, efficiency of CYP-redox partner interactions, the NAD(P)H regeneration system and the substrate transportation. Since various efforts have been made in optimizations, a combination of different methods could result in a higher yield. For example, with *E. coli* as the host, not only by expression of high enzymatic activity CYPs which are assisted with adequate Fdr and Fdx, the introduction of GroEL/ES system along with the mutation of genes include *acrAB* and *tolC* [67], whose expression products compose a efflux pump system which play positive roles simultaneously. The deletion of *acrAB* and *tolC* in *E. coli* BL21star(DE3) works similar to the nisin treatment for *R. erythropolis*, which can increase in the intracellular substrate concentration. In addition, in the fermentation process, the addition of Fe^2+^ and 5-aminolevulinic acid (5-ALA) [68], which is precursor of heme can be adopted in most cases to ensure the functional expression of CYPs.

In addition to improving the transportation of substrates, there are other potential methods of improving VD_3_ bioconversion. CYPs displayed on the cell surface of *E. coli* [69, 70] will provide alternative solution for the transportation of VD_3_. In the meanwhile, this method could be also used for the screening of highly efficient VD_3_ hydroxylases. The fusion of CYPs and redox partners could construct a self-sufficient P450 [71], which is promising for future methods of VD_3_ bioconversion.

## Conclusions

Calcifediol and calcitriol have bright market prospects in the poultry farming and pharmaceutical industries. Compared with the traditional chemical synthesis, bioconversion of VD_3_ to calcifediol and calcitriol represents a promising and eco-friendly technology, and will contribute to the sustainable development goals of United Nations. Given that VD_3_ hydroxylase plays a key role in the bioconversion of VD_3_ to calcifediol and calcitriol, a series of CYPs have been modified by protein engineering to increase their catalysis of VD_3_ hydroxylation. In addition, the VD_3_ permeability and the electron transfer from NADH to VD_3_ hydroxylase are also of great importance in determining the rate of VD_3_ bioconversion. Therefore, the lipid II-targeting antibiotic nisin, redox partners and NAD(P)H regeneration systems are employed to further improve the VD_3_ bioconversion by enhancing the VD_3_ uptake and optimizing the electron transfer pathway. High-throughput screening technology has shown its advantages in the field of strain screening [72], and high-throughput screening based on the calcifediol/calcitriol sensor can be used to screen microbial strains that are capable of converting VD_3_ to calcifediol or calcitriol more efficiently.

With increasing pressure to conserve energy and protect the environment, increasing attention is being paid to the bioproduction of value-added chemicals in a more eco-friendly manner [73, 74]. The “next generation industrial biotechnology” based on moderately halophilic bacteria, which can avoid sterilization and the use of fresh water, exhibits advantages over the current industrial biotechnology. Given that a large number of VD_3_-hydroxylating strains belong to actinomycetes, efficient screening of halophilic actinomycetes could be a promising strategy to obtain more ideal calcifediol/calcitriol-producing strains. In addition, engineering of the well-studied halophilic bacteria such as *Halomonas bluephagenesis* could be another promising strategy to further improve the calcifediol/calcitriol production in the future.

## Data Availability

Not applicable.

## Abbreviations

VD_3_: Vitamin D_3_
CYPs: Cytochrome P450s
S25DH: Steroid C25 dehydrogenase
